# Supplementary material for: A U1–U3 snRNA–snoRNA interaction couples SF3B1 mutation to chromatin-state rewiring and genome instability
Source: bioRxiv. 2026 Jun 8:2026.06.06.722969. Preprint. [Version 1] doi: 10.64898/2026.06.06.722969 (PMC13277980; doi:10.64898/2026.06.06.722969)
Supplement: Supplementary file 1 [file media-1.pdf]

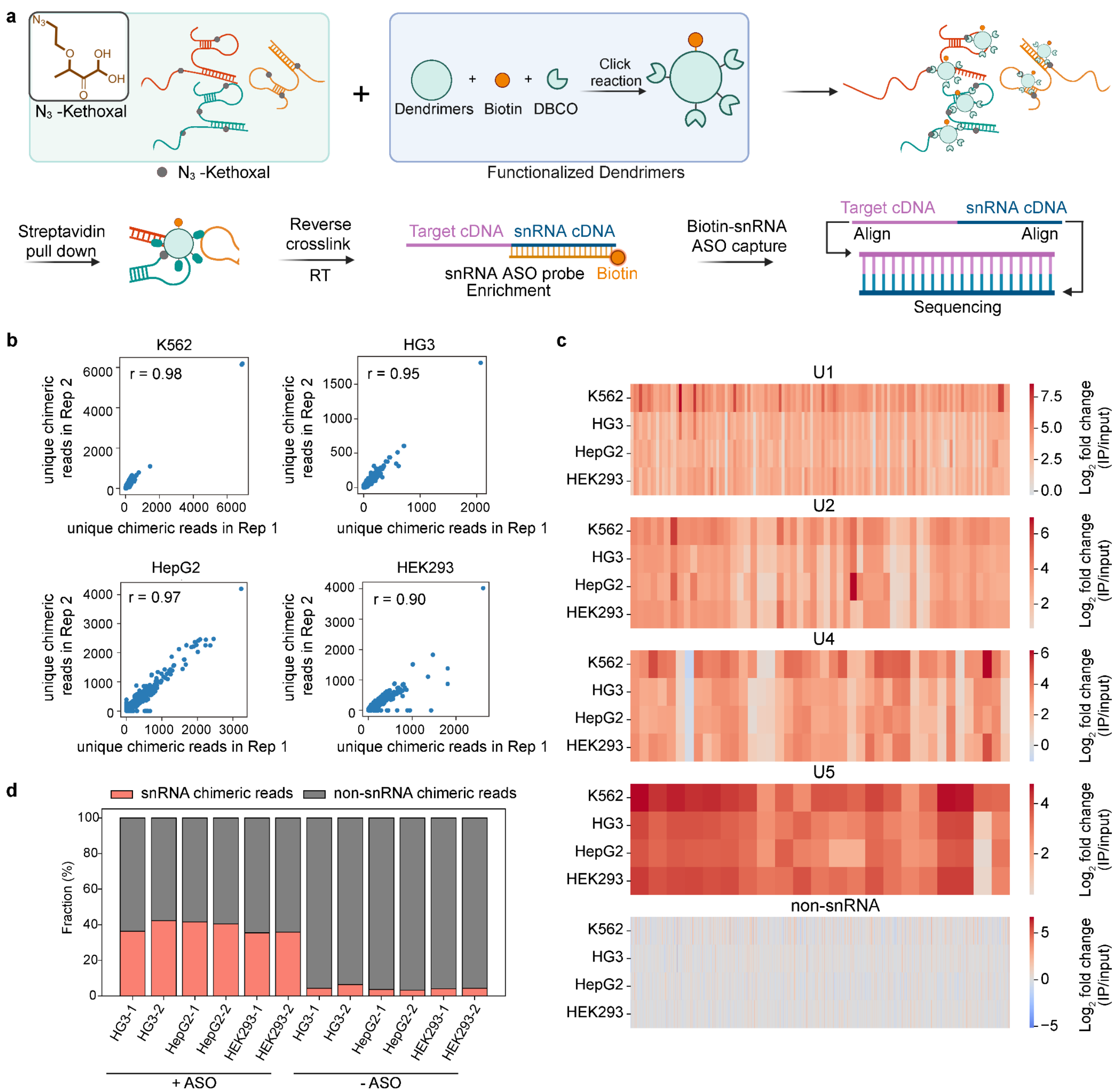

Extended Data Fig. 1 | snRNA enrichment and binding preferences.

a, Workflow schematic of the snRNA-enrichment KARR-seq (snRNA-KARR-seq) method. **b**, Correlation of unique chimeric reads obtained from biological replicates across K562, HG3, HepG2, and HEK293 cells. **c**, Enrichment efficiency of canonical snRNAs (U1, U2, U4, and U5) and non-snRNA total reads across K562, HG3, HepG2, and HEK293 cells. **d**, Efficiency of enrichment for chimeric reads involving U1, U2, U4, and U5 across K562, HG3, HepG2, and HEK293 cells.

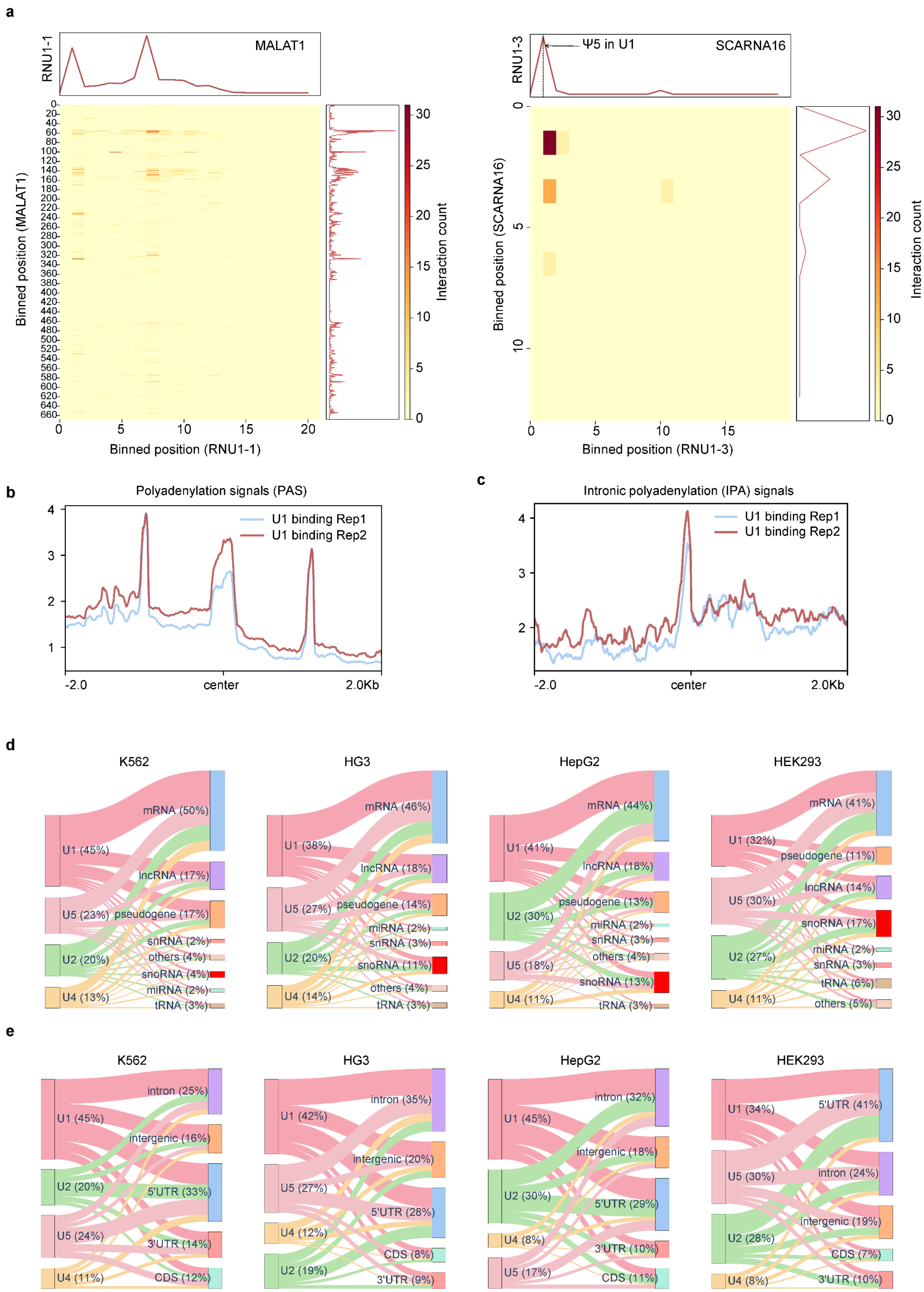
a
RNU1-1
MALAT1
Binned position (MALAT1)
Binned position (RNU1-1)
Interaction count
RNU1-3
Ψ5 in U1
SCARNA16
Binned position (SCARNA16)
Binned position (RNU1-3)
Interaction count
b
Polyadenylation signals (PAS)
U1 binding Rep1
U1 binding Rep2
-2.0
center
2.0Kb
c
Intronic polyadenylation (IPA) signals
U1 binding Rep1
U1 binding Rep2
-2.0
center
2.0Kb
d
K562
U1 (45%)
U5 (23%)
U2 (20%)
U4 (13%)
mRNA (50%)
lncRNA (17%)
pseudogene (17%)
snRNA (2%)
others (4%)
snoRNA (4%)
miRNA (2%)
tRNA (3%)
HG3
U1 (38%)
U5 (27%)
U2 (20%)
U4 (14%)
mRNA (46%)
lncRNA (18%)
pseudogene (14%)
miRNA (2%)
snRNA (3%)
snoRNA (11%)
others (4%)
tRNA (3%)
HepG2
U1 (41%)
U2 (30%)
U5 (18%)
U4 (11%)
mRNA (44%)
lncRNA (18%)
pseudogene (13%)
miRNA (2%)
snRNA (3%)
others (4%)
snoRNA (13%)
tRNA (3%)
HEK293
U1 (32%)
U5 (30%)
U2 (27%)
U4 (11%)
mRNA (41%)
pseudogene (11%)
lncRNA (14%)
snoRNA (17%)
miRNA (2%)
snRNA (3%)
tRNA (6%)
others (5%)
e
K562
U1 (45%)
U2 (20%)
U5 (24%)
U4 (11%)
intron (25%)
intergenic (16%)
5'UTR (33%)
3'UTR (14%)
CDS (12%)
HG3
U1 (42%)
U5 (27%)
U4 (12%)
U2 (19%)
intron (35%)
intergenic (20%)
5'UTR (28%)
CDS (8%)
3'UTR (9%)
HepG2
U1 (45%)
U2 (30%)
U4 (8%)
U5 (17%)
intron (32%)
intergenic (18%)
5'UTR (29%)
3'UTR (10%)
CDS (11%)
HEK293
U1 (34%)
U5 (30%)
U2 (28%)
U4 (8%)
5'UTR (41%)
intron (24%)
intergenic (19%)
CDS (7%)
3'UTR (10%)

Extended Data Fig. 2 | U1 snRNA enrichment and binding preferences.

a, 2D-interaction heatmaps illustrating known U1–MALAT1 (left) and U1–SCARNA16 (right) RNA interactions in K562 cells. **b**–**c**, U1 snRNA occupancy around polyadenylation signal (PAS) site (**b**) and intronic polyadenylation (IPA) site (**c**) in K562 cells. **d**, Sankey diagrams depicting the distribution of snRNA target classes across K562, HG3, HepG2, and HEK293 cells. **e**, Sankey diagrams showing the proportion and identity of mRNA/pe-mRNA targets across K562, HG3, HepG2, and HEK293 cells.

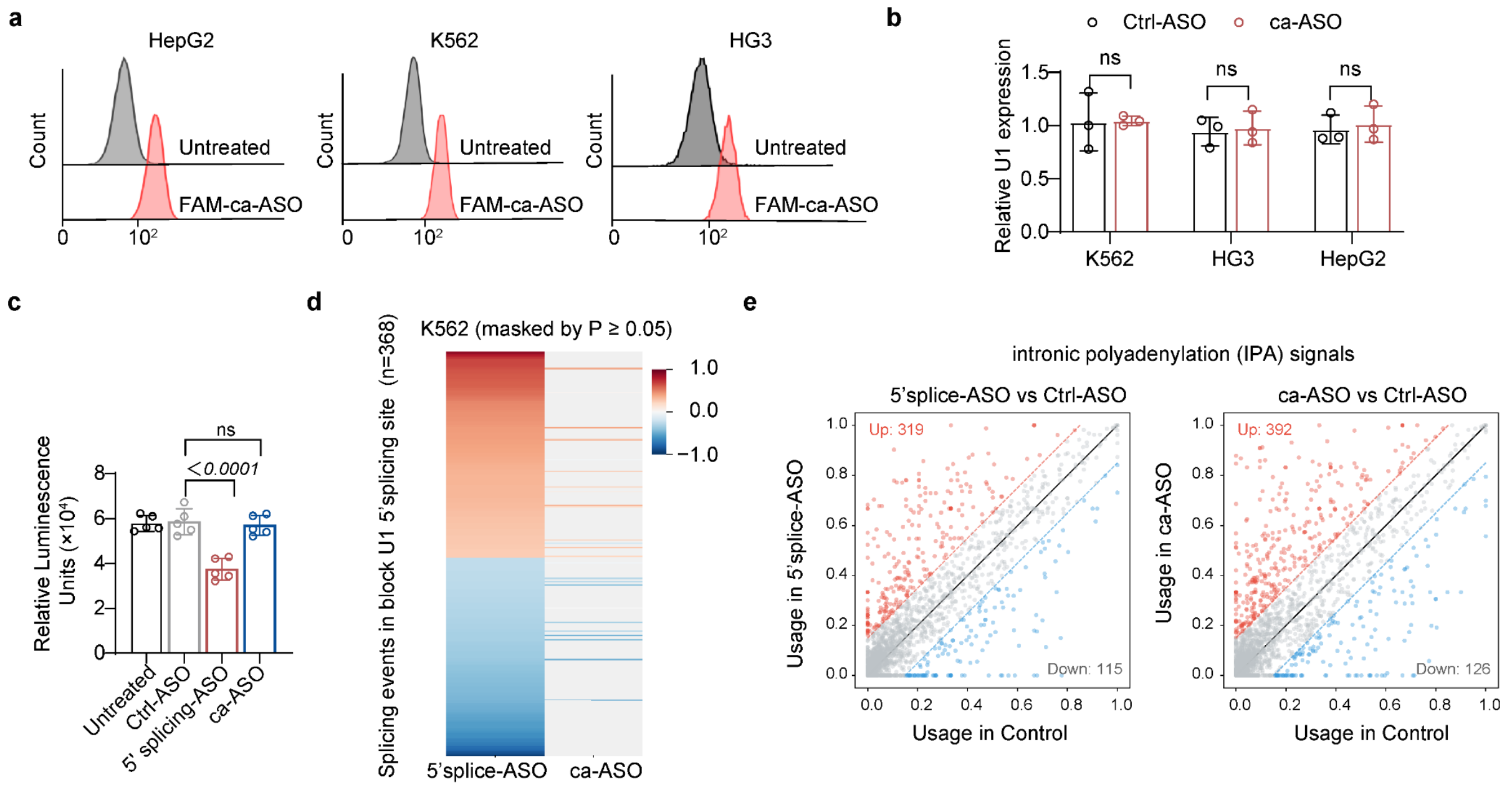

Extended Data Fig. 3 | Validation of ca-ASO activity and specificity.

a, Flow cytometry confirmed efficient uptake of FAM-labeled ca-ASO in HepG2, K562, and HG3 cells (n = 3 biological per group). **b**, qRT–PCR analysis of U1 snRNA expression in K562, HG3, and HepG2 cells following ca-ASO treatment (n = 3 biological per group). **c**, In vitro splicing of biotin-labelled Ad2ΔIVS pre-mRNA in whole-cell extract in the presence of the indicated ASOs, with exon junction complex (EJC) deposition quantified by HRP-based chemiluminescence (n = 5 biological per group). **d**, Heat map of differential splicing changes in K562 cells following transfection with the indicated ASOs. Events are ordered by direction and magnitude of change; non-significant values are masked ($P \geq 0.05$; n = 3 biological per group). **e**, Scatter plots of changes in intronic polyadenylation (IPA) site usage in K562 cells transfected with the indicated ASOs relative to control conditions (n = 3 biological per group). Data are mean ± s.d for **b** and **c**. P value was determined using an unpaired two-tailed t test (**b** and **c**). ns indicates no statistically significant difference.

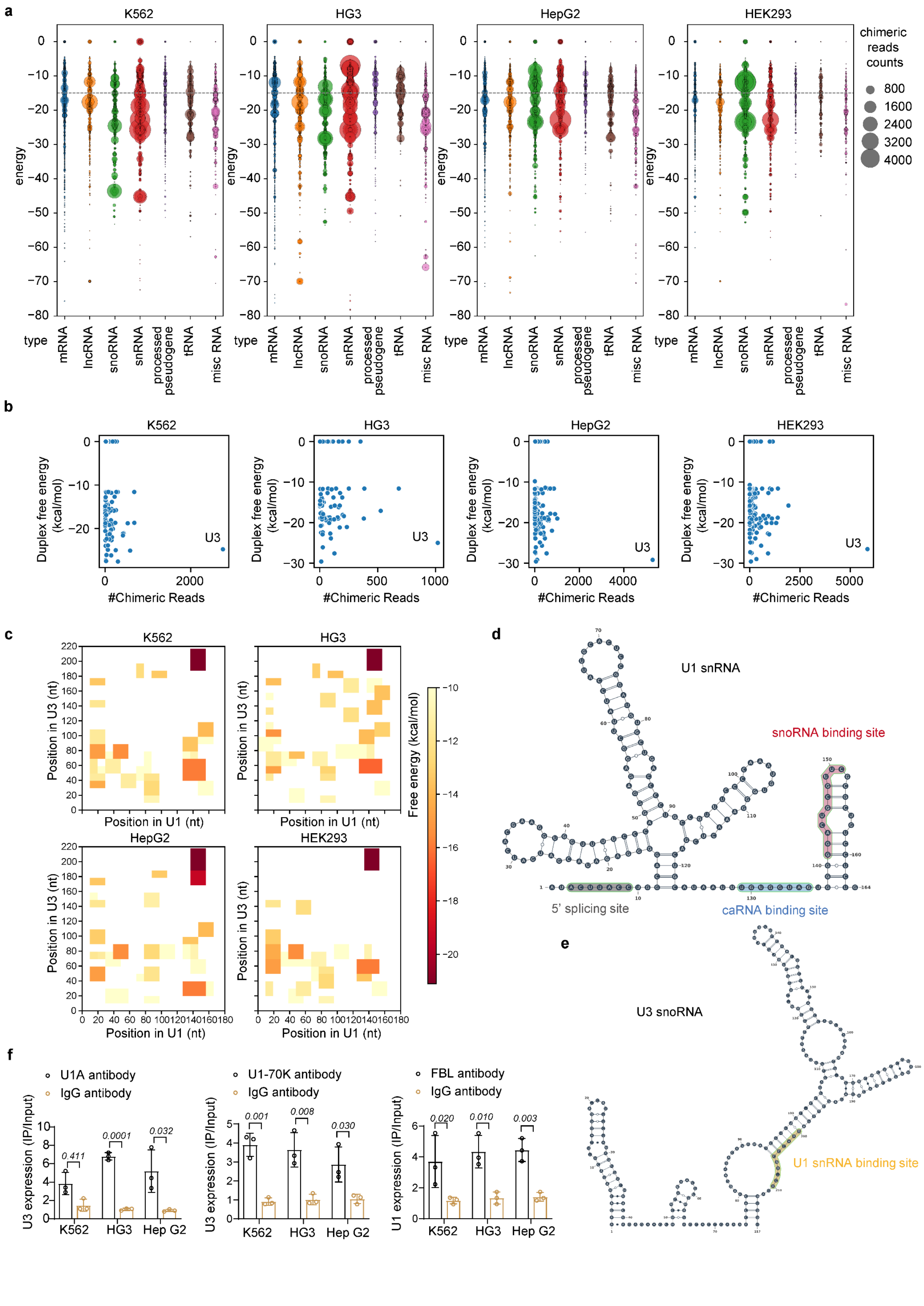
a
K562
HG3
HepG2
HEK293
chimeric reads counts
800
1600
2400
3200
4000
energy
type
mRNA
lncRNA
snoRNA
snRNA
processed pseudogene
tRNA
misc RNA
b
Duplex free energy (kcal/mol)
#Chimeric Reads
U3
c
Position in U3 (nt)
Position in U1 (nt)
Free energy (kcal/mol)
d
U1 snRNA
snoRNA binding site
5' splicing site
caRNA binding site
e
U3 snoRNA
U1 snRNA binding site
f
U1A antibody
IgG antibody
U1-70K antibody
FBL antibody
U3 expression (IP/Input)
U1 expression (IP/Input)
K562
HG3
Hep G2
0.411
0.0001
0.032
0.001
0.008
0.030
0.020
0.010
0.003

Extended Data Fig. 4 | KARR-seq analysis of snRNA–RNA interactions.

a, Predicted base pairing free energy values of snRNA–target duplexes across K562, HG3, HepG2, and HEK293 cells. **b**, Comparative analysis of chimeric read counts and predicted ΔG values for diverse snRNA-target interactions identified via KARR-seq across K562, HG3, HepG2, and HEK293 cells. **c**, Heatmap displaying predicted ΔG values of U1-U3 interactions across K562, HG3, HepG2, and HEK293 cells. **d**, U1 secondary structure model with color-coded binding motifs: splice site (grey), caRNA binding site (blue), and U1-U3 interaction region (red). **e**, Secondary structure model of U3 snoRNA, highlighting the predicted U1 binding site in yellow. **f**, RIP–qPCR of chromatin fraction from K562, HG3, and HepG2 cells showed enrichment of U3 snoRNA using U1A or U1-70K antibodies compared with IgG controls, and enrichment of U1 snRNA using FBL antibody compared with IgG controls (n = 3 biological per group). Data are mean ± s.d for **f**. P value was determined using an unpaired two-tailed t test (**f**).

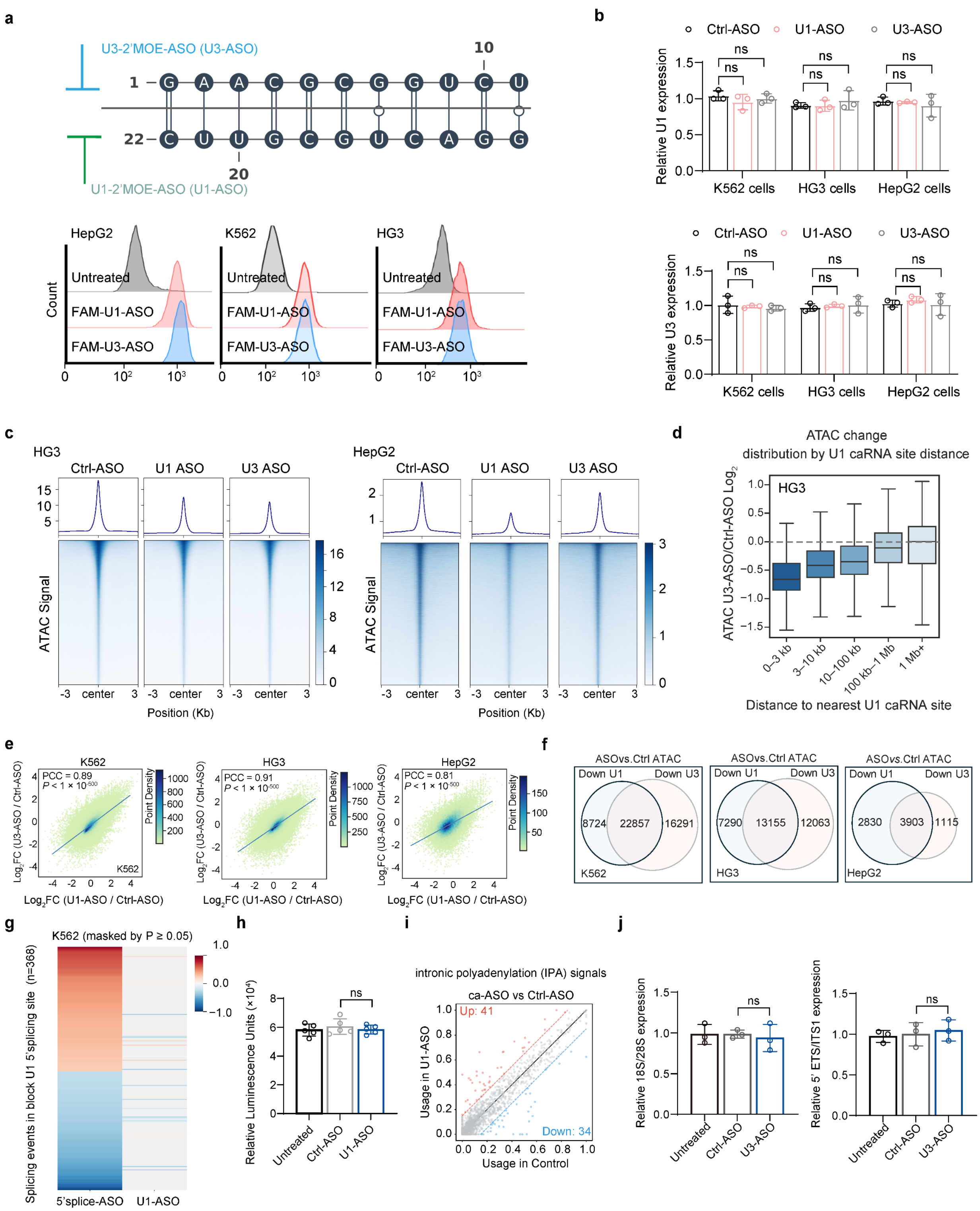

Extended Data Fig. 5 | Effects of U1-ASO and U3-ASO on chromatin accessibility.

a, Flow cytometry analysis of FAM-labelled ASO uptake in HepG2, K562, and HG3 cells (*n* = 3 biological per group). **b**, RT–qPCR quantification of U1 snRNA and U3 snoRNA abundance

following ASO treatment in HepG2, K562, and HG3 cells (*n* = 3 biological per group). **c**, Average ATAC-seq profiles and heat maps of chromatin accessibility in K562 and HG3 cells following U1-ASO or U3-ASO treatment. **d**, ATAC-seq signal changes as a function of distance from U1–caRNA binding sites. **e**, Correlation analysis of differential ATAC-seq peaks following U1-ASO or U3-ASO treatment in K562, HG3, and HepG2 cells. **f**, Venn diagram showing the overlap of differential ATAC peaks in K562, HG3, and HepG2 cells. **g**, Heat map of differential splicing changes in K562 cells following transfection with the indicated ASOs. Events are ordered by direction and magnitude of change; non-significant values are masked ($P \geq 0.05$; *n* = 3 biological per group). **h**, In vitro splicing of biotin-labelled Ad2ΔIVS pre-mRNA in whole-cell extract in the presence of the indicated ASOs, with exon junction complex (EJC) deposition quantified by HRP-based chemiluminescence (*n* = 5 biological per group). **i**, Scatter plots of changes in intronic polyadenylation (IPA) site usage in K562 cells transfected with the indicated ASOs relative to control conditions (*n* = 3 biological per group). **j**, RT–qPCR quantification of mature rRNA (18S and 28S) and pre-rRNA (5′ETS and ITS1) levels in K562 cells treated with the indicated ASOs (*n* = 3 biological per group). For **d**, the box plots show the median (centre line), upper and lower quartiles (box limits) and 1–99% (whiskers). *P* value was determined using an unpaired two-tailed *t* test (**b**), and Pearson's correlation coefficient (PCC) was used to assess correlation (**e**). ns indicates no statistically significant difference. ns indicates no statistically significant difference.

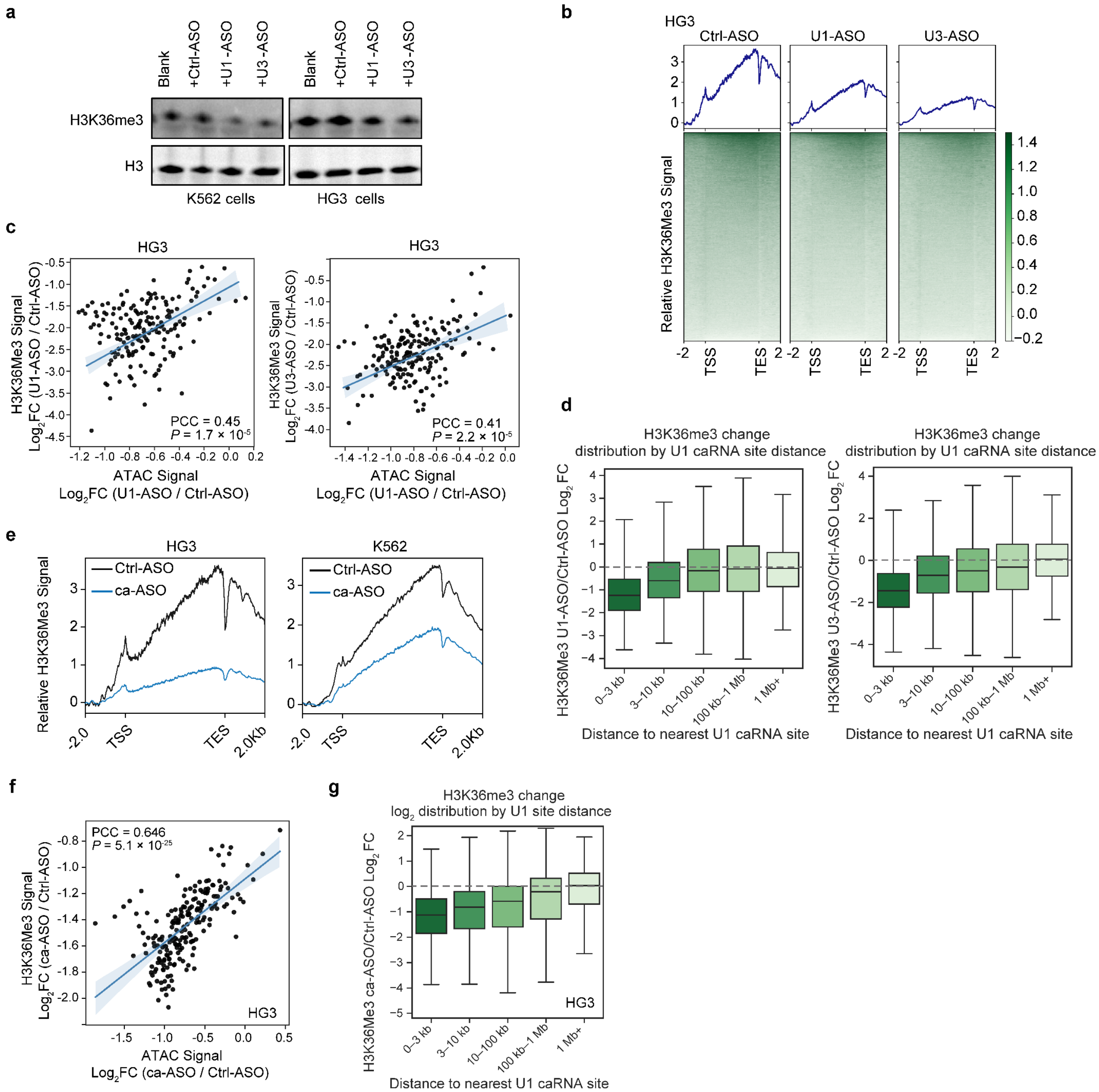

Extended Data Fig. 6 | Effects of U1-ASO and U3-ASO on histone modification.

a, Western blot analysis of H3K36me3 in K562 and HG3 cells following U1-ASO or U3-ASO treatment. **b**, CUT&Tag profiles showing changes in H3K36me3 peaks in HG3 cells after U1-ASO or U3-ASO treatment. **c**, Scatter plots showing the relationship between H3K36me3 signal changes and ATAC-seq signal changes in HG3 cells after U1–ASO treatment in HG3 cells. **d**, H3K36me3 changes in K562 cells displayed a clear spatial gradient, with the strongest reductions occurring within a few kilobases of U1–caRNA binding sites and progressively diminishing toward zero at more distal regions. **e**, CUT&Tag profiles of H3K36me3 in K562 and HG3 cells following ca-ASO treatment. **f**, Scatter plots showing the relationship between

H3K36me3 signal changes and ATAC-seq signal changes after ca–ASO treatment in HG3 cells. **g**, H3K36me3 changes displayed a clear spatial gradient, with the strongest reductions occurring within a few kilobases of U1–caRNA binding sites and progressively diminishing toward zero at more distal regions in HG3 cells. *n* = 3 biological per group. For **d** and **g**, the box plots show the median (centre line), upper and lower quartiles (box limits) and 1–99% (whiskers). Pearson's correlation coefficient (PCC) was used to assess correlation (**c** and **f**).

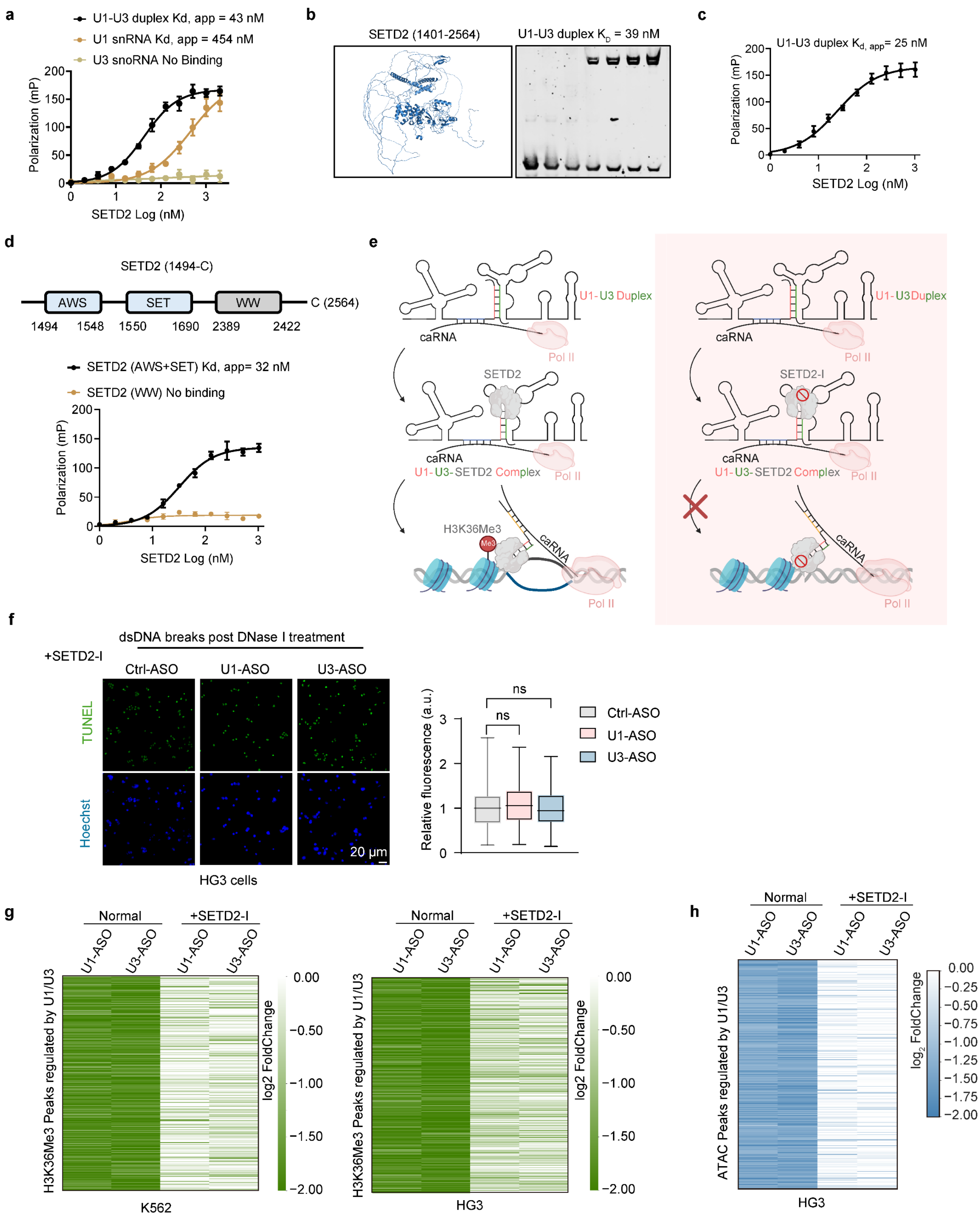

Extended Data Fig. 7 | SETD2 dependence of U1–U3–mediated chromatin regulation.
a, Fluorescence polarization assay comparing SETD2 binding to U1 snRNA, U3 snoRNA, and the U1–U3 duplex. **b**, Electrophoretic mobility shift assays showing direct binding of recombinant SETD2 truncation mutants to U1 snRNA, U3 snoRNA, and the U1–U3 duplex. **c**,

Fluorescence polarization assay measuring the binding of the U1–U3 RNA duplex to truncated SETD2. **d**, Domain organization of the SETD2 C-terminal region (amino acids 1494–2564), indicating the AWS, SET and WW domains (left), and fluorescence polarization assays measuring binding of the U1–U3 duplex to SETD2 fragments containing AWS+SET or the isolated WW domain (right). **e**, Schematic illustrating that inhibition of SETD2 prevents deposition of H3K36me3 at U1–U3 duplex–recruited sites. **f**, Representative DNase I and TUNEL staining in HG3 cells showing chromatin changes after SETD2 inhibition (left), with quantification across six fields of view (right). **g**, Changes of H3K36me3 signals in K562 and HG3 cells reduced by U1-ASO or U3-ASO before and after SETD2 inhibition. **h**, Changes in ATAC-seq peaks reduced by U1-ASO or U3-ASO before and after SETD2 inhibition in HG3 cells. $n = 3$ biological per group. For **f**, the box plots show the median (centre line), upper and lower quartiles (box limits) and 1–99% (whiskers). Data are mean ± s.d (**a**, **c**, and **d**). *P* value was determined using an unpaired two-tailed *t* test (**f**). TUNEL intensity was quantified by ImageJ.

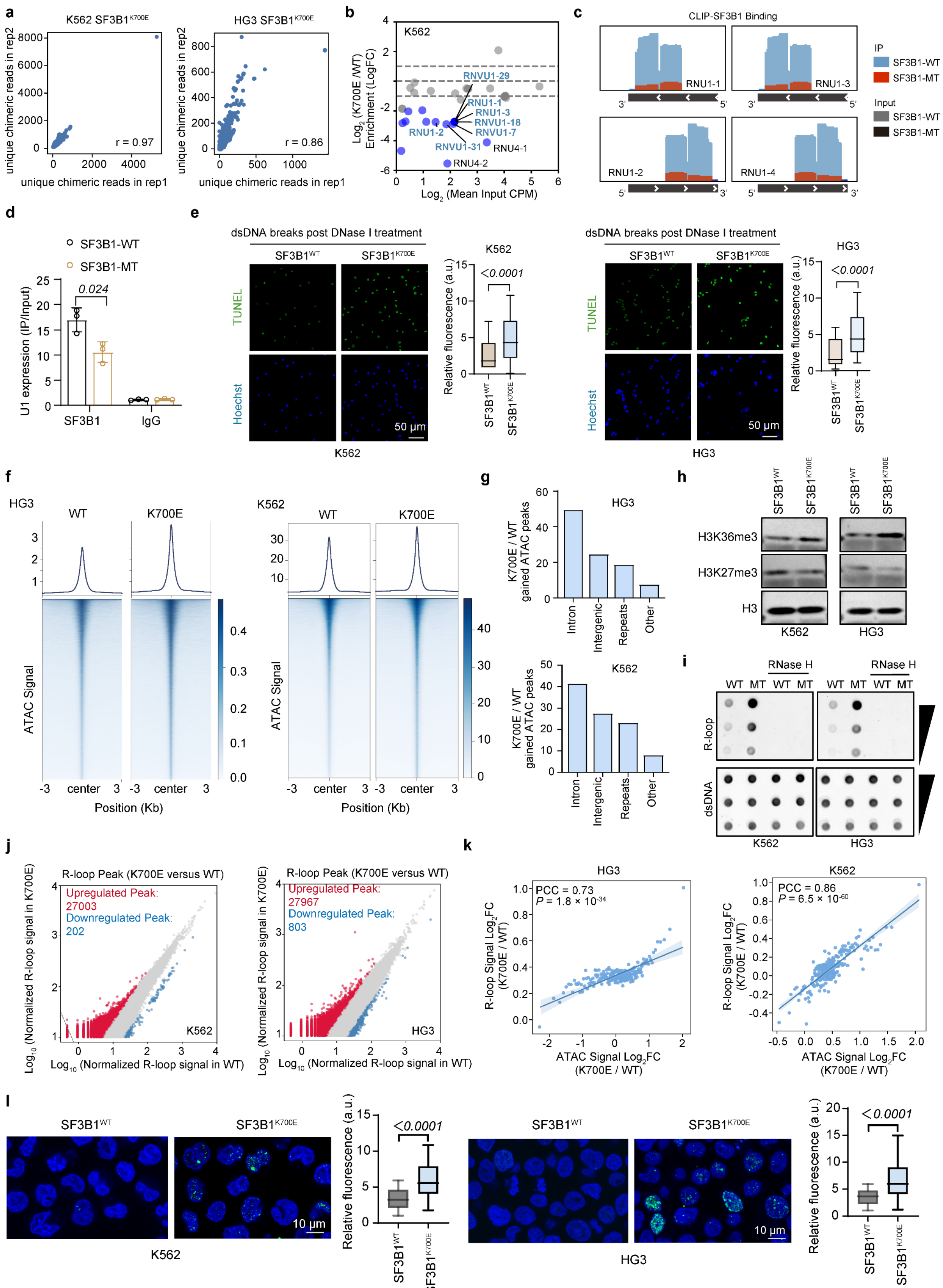

a
K562 SF3B1K700E
HG3 SF3B1K700E
unique chimeric reads in rep2
unique chimeric reads in rep1
r = 0.97
r = 0.86
b
K562
Log2 (K700E /WT) Enrichment (LogFC)
Log2 (Mean Input CPM)
RNVU1-29
RNU1-1
RNU1-3
RNVU1-18
RNVU1-7
RNU1-2
RNVU1-31
RNU4-1
RNU4-2
c
CLIP-SF3B1 Binding
RNU1-1
RNU1-3
RNU1-2
RNU1-4
IP
SF3B1-WT
SF3B1-MT
Input
SF3B1-WT
SF3B1-MT
d
SF3B1-WT
SF3B1-MT
U1 expression (IP/Input)
0.024
SF3B1
IgG
e
dsDNA breaks post DNase I treatment
SF3B1WT
SF3B1K700E
TUNEL
Hoechst
50 μm
K562
HG3
Relative fluorescence (a.u.)
<0.0001
f
HG3
K562
WT
K700E
ATAC Signal
center
Position (Kb)
g
HG3
K562
K700E / WT gained ATAC peaks
Intron
Intergenic
Repeats
Other
h
SF3B1WT
SF3B1K700E
H3K36me3
H3K27me3
H3
K562
HG3
i
RNase H
WT
MT
R-loop
dsDNA
K562
HG3
j
R-loop Peak (K700E versus WT)
Upregulated Peak: 27003
Downregulated Peak: 202
Upregulated Peak: 27967
Downregulated Peak: 803
K562
HG3
Log10 (Normalized R-loop signal in K700E)
Log10 (Normalized R-loop signal in WT)
k
HG3
K562
PCC = 0.73
P = 1.8 × 10-34
PCC = 0.86
P = 6.5 × 10-60
R-loop Signal Log2FC (K700E / WT)
ATAC Signal Log2FC (K700E / WT)
l
SF3B1WT
SF3B1K700E
10 μm
K562
HG3
Relative fluorescence (a.u.)
<0.0001

Extended Data Fig. 8 | Chromatin alterations and genome instability in SF3B1-mutant cells.

a, Correlation of unique chimeric reads obtained from biological replicates across HG3 $SF3B1^{K700E}$ and K562 $SF3B1^{K700E}$ cell lines. **b**, CLIP–seq analysis showing differential snRNA binding in K562 $SF3B1^{K700E}$ (MT) versus K562 $SF3B1^{K700K}$ (WT) cells. Scatter plot displays log_2 enrichment changes (MT/WT) relative to mean input abundance for individual snRNAs. Blue points indicate snRNAs with reduced association in mutant cells. **c**, CLIP-seq using anti-Flag antibody showing RNA binding profiles of K562 $SF3B1^{K700K}$ and $SF3B1^{K700E}$ cells, respectively, with IGV tracks depicting U1-binding sites. **d**, RIP-qPCR showing enrichment of U1 snRNA by Flag antibody in K562 $SF3B1^{WT}$ and $SF3B1^{K700E}$ cells. **e**, Representative DNase I and TUNEL staining in $SF3B1^{WT}$ and $SF3B1^{K700E}$ K562 cells as well as HG3 cells (left), with quantification across six fields of view (right). **f**, Average ATAC-seq profiles and heat maps of chromatin accessibility in $SF3B1^{WT}$ and $SF3B1^{K700E}$ K562 cells as well as HG3 cells. **g**, Genomic distribution of ATAC-seq peaks gained in HG3 and K562 $SF3B1^{K700E}$ cells versus HG3 and K562 $SF3B1^{WT}$ cells. **h**, Western blot showing H3K36me3 and H3K27me3 differences between $SF3B1^{WT}$ and $SF3B1^{K700E}$ K562 and HG3 cells. **i**, Dot blot showing R-loop levels in $SF3B1^{WT}$ and $SF3B1^{K700E}$ K562 and HG3 cells. **j**, Correlation between differential R-loop peaks and ATAC-seq peaks in $SF3B1^{WT}$ and $SF3B1^{K700E}$ K562 and HG3 cells. **k**, Correlation between changes in chromatin accessibility and R-loop abundance in SF3B1-K700E K562 and HG3 cells. Scatter plots show the relationship between ATAC-seq log_2 fold change and R-loop signal log_2 fold change in HG3 (left) and K562 (right) cells. **l,** Representative images and quantification of phosphorylated γ-H2AX foci in $SF3B1^{WT}$ and $SF3B1^{K700E}$ K562 and HG3 cells. Quantification was performed across six fields of view. $n = 3$ biological per group. Data are mean ± s.d for **d**. *P* value was determined using an unpaired two-tailed *t* test (**d**, **e**), and Pearson's correlation coefficient (PCC) was used to assess correlation (**k**). Signal intensity was measured with ImageJ.

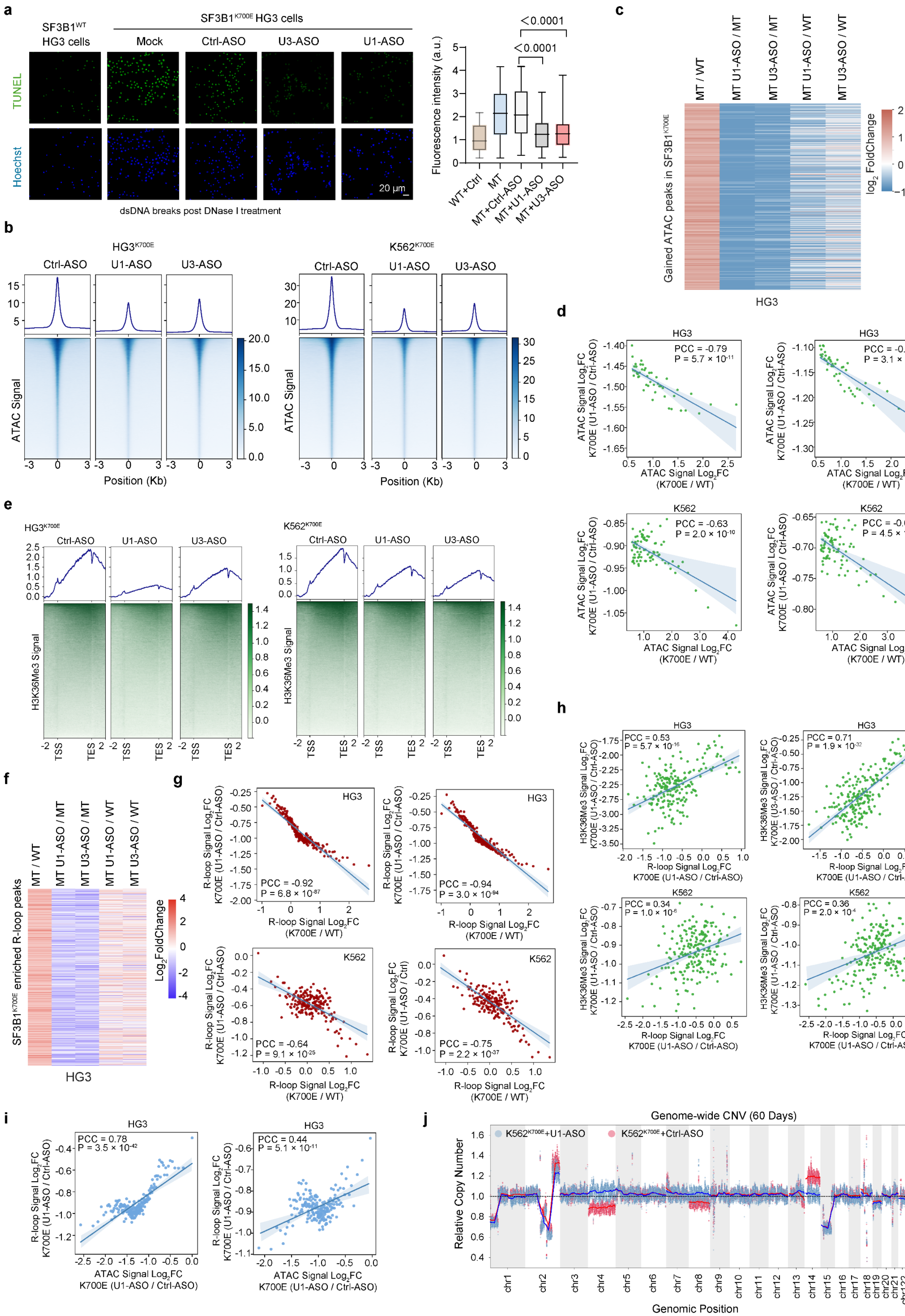
a
SF3B1WT
HG3 cells
SF3B1K700E HG3 cells
Mock
Ctrl-ASO
U3-ASO
U1-ASO
TUNEL
Hoechst
20 μm
dsDNA breaks post DNase I treatment
Fluorescence intensity (a.u.)
<0.0001
<0.0001
WT+Ctrl
MT
MT+Ctrl-ASO
MT+U1-ASO
MT+U3-ASO
b
HG3K700E
K562K700E
Ctrl-ASO
U1-ASO
U3-ASO
ATAC Signal
Position (Kb)
c
MT / WT
MT U1-ASO / MT
MT U3-ASO / MT
MT U1-ASO / WT
MT U3-ASO / WT
Gained ATAC peaks in SF3B1K700E
log2 FoldChange
HG3
d
HG3
PCC = -0.79
P = 5.7 × 10-11
PCC = -0.82
P = 3.1 × 10-12
K562
PCC = -0.63
P = 2.0 × 10-10
PCC = -0.62
P = 4.5 × 10-10
ATAC Signal Log2FC
K700E (U1-ASO / Ctrl-ASO)
ATAC Signal Log2FC
K700E (U3-ASO / Ctrl-ASO)
ATAC Signal Log2FC
(K700E / WT)
e
HG3K700E
K562K700E
Ctrl-ASO
U1-ASO
U3-ASO
H3K36Me3 Signal
TSS
TES
f
MT / WT
MT U1-ASO / MT
MT U3-ASO / MT
MT U1-ASO / WT
MT U3-ASO / WT
SF3B1K700E enriched R-loop peaks
Log2FoldChange
HG3
g
HG3
PCC = -0.92
P = 6.8 × 10-87
PCC = -0.94
P = 3.0 × 10-94
K562
PCC = -0.64
P = 9.1 × 10-25
PCC = -0.75
P = 2.2 × 10-37
R-loop Signal Log2FC
K700E (U1-ASO / Ctrl-ASO)
R-loop Signal Log2FC
K700E (U1-ASO / Ctrl)
R-loop Signal Log2FC
(K700E / WT)
h
HG3
PCC = 0.53
P = 5.7 × 10-16
PCC = 0.71
P = 1.9 × 10-32
K562
PCC = 0.34
P = 1.0 × 10-6
PCC = 0.36
P = 2.0 × 10-4
H3K36Me3 Signal Log2FC
K700E (U1-ASO / Ctrl-ASO)
H3K36Me3 Signal Log2FC
K700E (U3-ASO / Ctrl-ASO)
R-loop Signal Log2FC
K700E (U1-ASO / Ctrl-ASO)
i
HG3
PCC = 0.78
P = 3.5 × 10-42
PCC = 0.44
P = 5.1 × 10-11
R-loop Signal Log2FC
K700E (U1-ASO / Ctrl-ASO)
R-loop Signal Log2FC
K700E (U3-ASO / Ctrl-ASO)
ATAC Signal Log2FC
K700E (U1-ASO / Ctrl-ASO)
ATAC Signal Log2FC
K700E (U3-ASO / Ctrl-ASO)
j
Genome-wide CNV (60 Days)
K562K700E+U1-ASO
K562K700E+Ctrl-ASO
Relative Copy Number
chr1
chr2
chr3
chr4
chr5
chr6
chr7
chr8
chr9
chr10
chr11
chr12
chr13
chr14
chr15
chr16
chr17
chr18
chr19
chr20
chr21
chr122
Genomic Position

Extended Data Fig. 9 | U1–U3 disruption modulates chromatin accessibility, R-loop accumulation and genome instability in SF3B1^{K700E} cells.

a, Representative DNase I and TUNEL staining in HG3 SF3B1^{K700E} cells after U1-ASO or U3-ASO treatment (left), with quantification across six fields of view (right). TUNEL intensity was quantified by ImageJ. **b,** Average ATAC-seq profiles and heat maps of chromatin accessibility in K562 SF3B1^{K700E} and HG3 SF3B1^{K700E} cells following U1-ASO or U3-ASO treatment. **c,** Heat map illustrating the spike-in-calibrated ATAC–seq signals across ATAC-seq peak regions that are more accessible in HG3 SF3B1^{K700E} cells than in HG3 SF3B1^{WT} cells, and these signal changes in HG3 SF3B1^{K700E} cells following U1-ASO or U3-ASO treatment. **d,** Correlation analysis comparing ATAC-seq changes upon U1–U3 disruption (U1-ASO/Ctrl-ASO) with ATAC-seq alterations observed in SF3B1^{K700E} versus SF3B1^{WT} K562 and HG3 cells. **e,** CUT&Tag profiles of H3K36me3 following U1-ASO or U3-ASO treatment in SF3B1^{K700E} K562 and HG3 cells. **f,** Heatmap showing spike-in–calibrated R-loop signals at R-loop peak regions that are increased in HG3 SF3B1^{K700E} cells compared with HG3 SF3B1^{WT}cells, and their changes following U1-ASO or U3-ASO treatment. **g,** Correlation analysis comparing R-loop signal changes upon U1–U3 disruption (U1-ASO/Ctrl-ASO) with R-loop alterations observed in SF3B1^{K700E} versus SF3B1^{WT} K562 and HG3 cells. **h,** Correlation analysis comparing H3K36me3 changes induced by U1–U3 disruption (U1-ASO/Ctrl-ASO) with corresponding R-loop alterations in SF3B1^{K700E} versus SF3B1^{WT} K562 and HG3 cells. **i,** Correlation between peaks showing reduced ATAC-seq signal and reduced R-loop accumulation after ASO treatment in HG3 SF3B1^{K700E} cells. **j,** Whole-genome sequencing (WGS) analysis of copy-number variation (CNV) in K562 SF3B1^{K700E} cells 60 days after U1-ASO or Ctrl-ASO treatment. CNV profiles were derived from WGS read-depth data and normalized to Ctrl-ASO–treated K562 SF3B1^{K700K} data. For **a**, the box plots show the median (centre line), upper and lower quartiles (box limits) and 1–99% (whiskers). Pearson's correlation coefficient (PCC) was used to assess correlation (**d**, **g**, **h** and **i**). *P* value was determined using an unpaired two-tailed *t* test (**a**). TUNEL intensity was quantified by ImageJ.

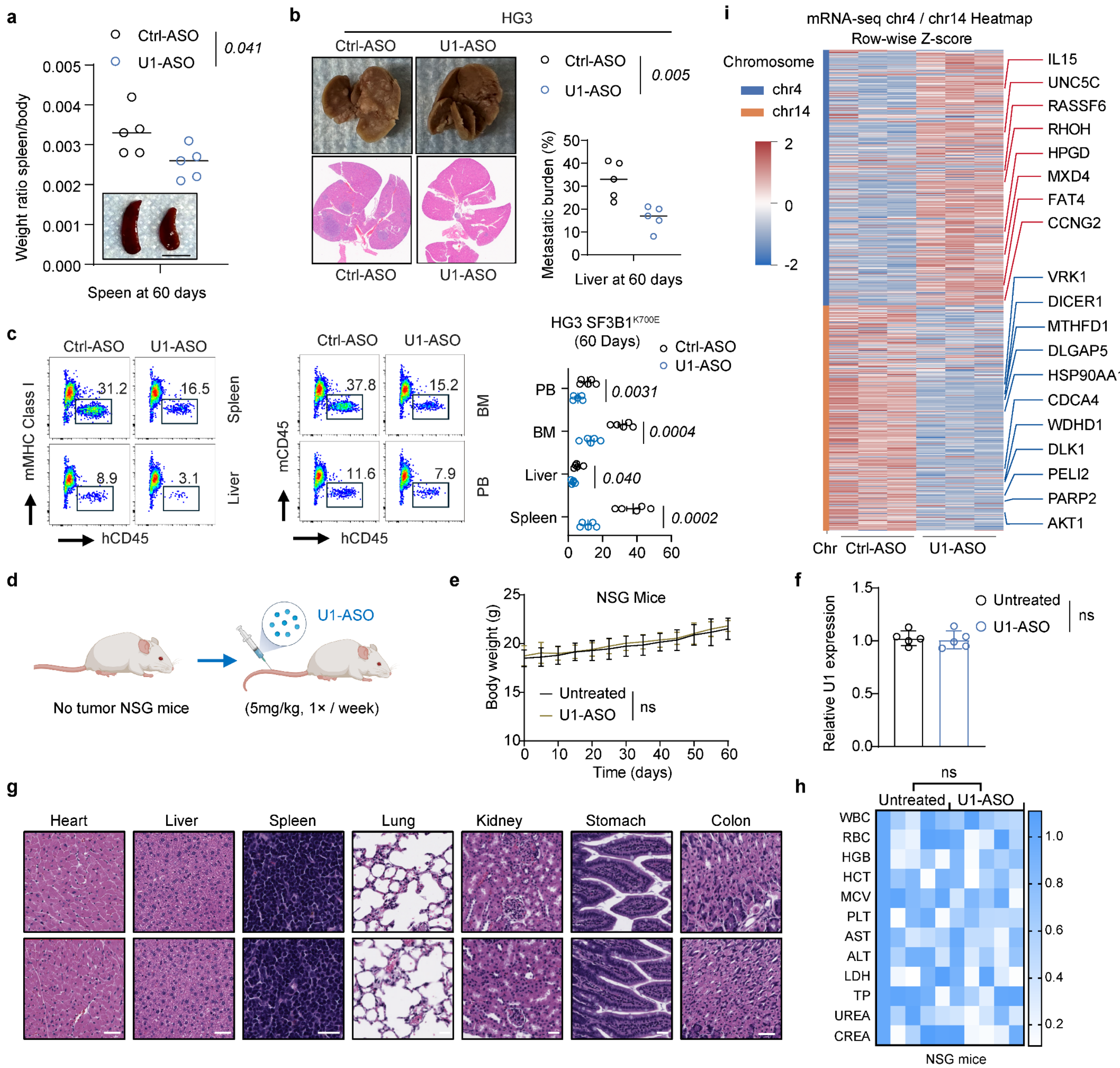

Extended Data Fig. 10 | In vivo effects and safety of U1-ASO treatment.
a, Representative images showing spleen size in control-ASO– and U1-ASO–treated mice bearing HG3 SF3B1^{K700E} xenografts. Quantification of spleen weight normalized to body weight is shown on the top. **b**, Number of hepatic metastatic foci in control-ASO– and U1-ASO–treated mice bearing HG3 SF3B1^{K700E} xenografts. **c**, Representative flow cytometry plots showing human leukemia cell (HG3 SF3B1^{K700E}) infiltration in peripheral blood, bone marrow, and solid organs. **d**, Schematic of the experimental design for evaluating U1-ASO safety in mice. **e**, Body weight trajectories of NSG mice treated with U1-ASO or left untreated over the indicated time course. **f**, Relative U1 snRNA expression in mouse peripheral blood measured by RT–qPCR in untreated and U1-ASO–treated mice. **g**, Representative haematoxylin and eosin (H&E) staining of major organs (heart, liver, spleen, lung, kidney, stomach and colon; scale bar, 50 μm). **h**, Safety analysis of untreated and U1-ASO–treated mice, showing blood biochemistry and liver and kidney function. **i**, Heat map of differential mRNA expression from chromosomes 4 and 14

in xenograft-derived K562 SF3B1^{K700E} cells, shown as row-wise z-scores. Representative upregulated and downregulated genes are highlighted with red and blue labels, respectively. n = 5 biological per group. Data are mean ± s.d. P value was determined using an unpaired two-tailed t test (**a**, **b**, **c**, **f** and **h**) or two-way ANOVA with Tukey's post-test (**e**). ns indicates no statistically significant difference.
